# Spectral Studies and Bactericidal, Fungicidal, Insecticidal and Parasitological Activities of Organotin(IV) Complexes of Thio Schiff Bases Having no Donor Atoms

**DOI:** 10.1155/MBD.1995.297

**Published:** 1995

**Authors:** Mala Nath, Savita Goyal

**Affiliations:** 1 Department of Chemistry University of Roorkee Roorkee 247667 (U.P.) India

## Abstract

Twelve new organotin(IV) complexes of the type RnSnLm  [where n = 3, m = 1, R = CH_3_ or C_6_H_5_;  n = 2, m =  2, R = C_6_H_5_ or C_4_H_9_ ; L = anion of Schiff bases derived from the condensation of 2-amino-5-(*o*-anisyl)-l,3,4-thiadiazole with salicylaldehyde (HL-1), 2- hydroxynaphthaldehyde (HL-2) and 2-hydroxyacetophenone (HL-3)] have been synthesized and characterized by elemental analysis, molar conductances, electronic, infrared, far-infrared, ^1^H NMR and ^119^Sn Mössbauer spectral studies. Thermal studies of two complexes, viz., Ph_3_Sn (L-1) and Ph_2_Sn(L-2)_2_ have been carried out in the temperature range 25-1000∘C  using TG, DTG and DTA techniques. All these complexes decompose gradually with the formation of SnO_2_ as an end product. In *vitro* antimicrobial activity of the Schiff bases and their complexes has also been determined against *Streptococcus faecalis, Klebsiella pneumoniae, Escherichia coli, Pseudomonas aeruginosa, Staphylococcus aureus* Penicillin resistance (2500 units), *Candida albicans, Cryptococcus neoformans, Sporotrichum schenckii, Trichophyton mentagrophytes and Aspergillus fumigatus*. The Schiff bases (HL-1), (HL-2) and the organotin(IV) compounds have also been tested against various important herbicidal, fungicidal, insecticidal species and also for parasitological activity against freeliving nematode.


					SPECTRAL STUDIES AND BACTERICIDAL, FUNGICIDAL,
INSECTICIDAL AND PARASITOLOGICAL ACTIVITIES
OF ORGANOTIN(IV) COMPLEXES OF THIO SCHIFF BASES
HAVING NO DONOR ATOMS
Mala Nath* and Savita Goyal
Department of Chemistry, University of Roorkee, Roorkee 247667 (U.P.), India
ABSTRACT
Twelve new organotin(IV) complexes of the type R,SnL [where n 3, rn 1, R CH or
C6H n 2, rn 2, R C6H or C4H9
L anion of Schiff bases derived from the conden-
sation of 2-amino-5-(o-anisyl)-l,3,4-thiadiazole with salicylaldehyde (HL-1), 2-
hydroxynaphthaldehyde (HL-2) and 2-hydroxyacetophenone (HL-3)] have been synthesized and
characterized by elemental analysis, molar conductances, electronic, infrared, far-infrared, H NMR
and gSn M6ssbauer spectral studies. Thermal studies of two complexes, viz., PhsSn(L-1) and
PhzSn(L-2)2 have been carried out in the temperature range 25-1000C using TG, DTG and DTA
techniques. All these complexes decompose gradually with the formation of SnOz as an end product.
In vitro antimicrobial activity of the Schiff bases and their complexes has also been determined
against Streptococcus faecalis, Klebsiella pneumomae, Escherichia co#, Pseudomonas aeruginosa,
Staphylococcus aureus Penicillin resistance (2500 units), Candida albicans, Cryptococcus
neoformans, Sporotrichum schenckii, Trichophyton mentagrophytes and Aspergillus fumigatus. The
Sehiff bases (HL-1), (HL-2) and the organotin(IV) compounds have also been tested against various
important herbicidal, fungicidal, insecticidal species and also for parasitological activity against
freeliving nematode.
INTRODUCTION
2,5-Disubstituted-l,3,4-thiadiazole moieties have been found to possess herbicidal, radioprotective,
diuretic and bacteriostatic properties (1-3). However, very little work seems to have been done
on the Schiff base complexes containing heterocyclic amines. Therefore, we report herein the syn-
thesis and characterization of organotin(IV) complexes with the Schiff bases described in Fig.l,
obtained by the condensation of 2-amino-5-(o-anisyl)-l,3,4-thiadiazole with aldehydes or ketones.
MATERIALS AND METHODS
All the reagents, viz., organotin halides (Fluka), salicylaldehyde, 2-hydroxynaphthaldehyde and 2-
hydroxyacetophenone (Fluka) were used as received. All the chemicals and solvents used were
dried and purified by standard methods, and moisture was excluded from the glass apparatus us-
ing CaCI: drying tubes.
Carbon, hydrogen and tin content of th complexes were determined as previously rported
(4). Molar conductance, electronic spectra, infrared, far-infrared, H NMR, gSn M6ssbauer and
thermal measurements were carried out on the sam instruments as previously reported (5).
Antimicrobial activities of the Schiff bases and some of their organotin(IV) complexes were
carried out at microbial section of the Central Drug Research Institute (CDRI), Lucknow using
two fold serial dilution techniques. Herbicide, insecticide and fungicide efficacy as well as para-
sitological activity of a few compounds were evaluated by Cyanamid, U.S.A.
Synthesis of the Schiff bases
2-Amino-5-(o-anisyl)-l,3,4-thiadiazole was prepared by oxidative cyclisation of 2-
297
VoL 2. No. 6. 1995 Spectral Studies & Bactericidal Fungicidal,Insecticidal
& ParasitologicalActivities ofOrganotin(IV) Complexes
methoxybenzaldehydethiosemicarbazone using ferric chloride hexa hydrate (6). The Schiff bases
were prepared by refluxing 2-amino-5-(o-anisyl)-l,3,4-thiadiazole (1 mole) and aldehyde or ket-
N----N
\
OCH3 R2
RI= H , R2 =
HL =
Where
R = CH3 R2 =
OH OH
(L-l) (L-2)
OH
(L-S)
Fig. l" General formula of the Schiff base.
tone (1 mole) in distilled ethanol (50 ml)) for 2 hrs. The coloured solid was obtained on cooling
after the removal of excess of solvent by distillation. It was recrystallized from the same solvent
and dried under vacuum.
Synthesis of the Complexes
The complexes were prepared by the replacement reactions of tri- and diorganotin(IV) chloride
with the sodium salts of the Schiff bases in 1"1 and 1.'2 ratios, respectively, in absolute alcohol
according to the folloxving method.
Sodium methoxide was prepared by the dissolution of sodium (0.16 g, 7.00 mmole) in ab-
solute methanol (10 ml). To this solution was added 5.00 mmole of the Schiff base [(HL-I) to
(HL-3)] in 25 ml of methanol and the mixture was first stirred for hr and then refluxed for 2
hrs. The sodium salt of the Schiff base so obtained in solution was added dropwise to the
triorganotin chloride (5.00 mmole) or diorganotin dichloride (2.50 mmole) in absolute methanol
(15 ml) with constant stirring. The resulting mixture was stirred for hr and then refluxed for
5-6 hrs. The contents were centrifuged to remove the sodium chloride and the unreacted sodio
derivative of the Schiff base. The excess solvent was removed by distillation. The complex thus
obtained was purified by recrystallization from petroleum ether (60-80C) and dried under vacuum.
1:2 1.1
Ph2SnCI2
/ RaSnCI + NaL > Ph2SnL: / R3SnL + 2NaCI / NaCI
Where R CH3
or C6H L anion of Schiff bases (HL-I) to (HL-3), as indicated in Fig. 1.
Reactions of di-n-butyltin oxide with the Schiff bases have been carried out in 1:2 molar
ratio in refluxing benzene. These reactions proceed with the liberation of water, which is re-
moved azeotropically with benzene.
Benzene-methanol
Bu2SnO+2HL .> Bu2SnL2+H20
Reflux for 8-10 hrs
Where L anion of Schiff bases (HL-I) to (HL-3)
298
M. Nath andS. Goyal Metal-BasedDrugs
299
Vol. o No. 6o 199.$ Spectral Studies & Bactericidal, Fungicidal,Insecticidal
&ParasitologicalActivities ofOrganotinV) Complexes
300
3/1. Nath andS. Goyal Metal-BasedDrugs
The above reactions were found to be quite facile and could be completed in 8-10 hrs of
refluxing. The resulting complexes are obtained in good yield in the form of coloured solids.
Results and Discussion
Stoichiometry
All the newly synthesized complexes are coloured solids and soluble in common organic solvents.
The colours, yields, melting points, elemental analyses and molar conductances of the Schiff bases
and their complexes are presented in Table 1. The analytical data are in good agreement with
the proposed stoichiometry of the complexes. The low values of the molar conductance (6.28-
13.85 ohm
-cm2
mole-) are in agreement with their non-electrolytic nature.
Electronic Spectra
In the electronic spectra of the Schiff bases and of their organotin(IV) complexes, two bands
are observed (Table II) in the region 217 233 nm and 270 309 nm which may be as-
Table I1" Electronic Spectral Bands (in nm) and their Assignments in the Schiff
Bases and their Complexes
Sl. Schiff base/ rt-Tt* 7-z* Charge trans-
No. Complex (benzenoid) (C=N) fer band from
iigand to tin
Secondary band of ben-
zene ring coupled with
intramolecular charge
transfer band
1. (HL-1) 223 293 372
2. Ph3Sn(L-1) 221 293 322 372
3. Me3Sn(L-1) 227 280 318,325 369
4. Ph2Sn(L-1)2 224 287 313,331 380
5. Bu2Sn(L-1)2 231 270 318 370
6. (HL-2) 219 309 355,428
7. PhaSn(L-2) 224 293sh 319 538
8. M%Sn(L-2) 233 272 319 538
9. Ph2Sn(L-2)2 230 295 318 489
10. Bu2Sn(L-2)2 228 288 321 410sh
11. (HL-3) 222 294sh 388
12. PhaSn(L-3) 217 290 328 397
13. MeSn(L-3) 233 298 325 388
14. Ph2Sn(L-3)2 227 277 313 334
15. Bu2Sn(L'3)2 224 296 318 337
sh, shoulder; a, in methanol.
signed to 7t-rt* transition of the benzenoid and of the >C--N chromophore, respectively. A
band in the region 355-538 nm in the spectra of the Schiff bases and complexes is likely to
301
Vol. 2, No. 6, 1995 Spectral Studies & Bactericidal, Fungicidal,Insecticidal
& ParasitologicalActivities ofOrganotin(IlO Complexes
be the secondary band of the benzene ring coupled with the intramolecular charge transfer transi-
tion taking place within the ligand moiety. Furthermore, sharp bands were observed in the region
313-331 nm in the spectra of the complexes which could be assigned to the charge transfer tran-
sition from ligand to tin (7).
Infrared Spectra
The characteristic infrared frequencies of the Schiff bases and their organotin(IV) complexes
are given in Table III. The infrared spectra of the Schiff bases exhibit a band at- 2940
cm
-characteristic of hydrogen bonded phenolic vOH vibrations (8). It is absent in the spec-
Table II1: Structurally Important IR Absorption Bands (cm") of the Schiff Bases and
their Complexes
Sl.* vC=N v >C vC-O vSn<---N vSn--O v,,, v,Sn-C
No. (azomethine) N-N=C<
1. 1632m 1600vs 1258m
2. 1622m 1598vs 1280vs 412vs 530vs 270s,235m
3. 1612m 1598vs 1265s 426m 505m 626m,570m
4. 1617m 1598vs 1273vs 424m 570m 262m,226m
5. 1620m 1598vs 1268vs 424s 568vs 606m,530m
6. 1628m 1596vs 1256s
7. 1610m 1595vs 1272vs 413vs 540s 271m,233s
8. 1609m 1595vs 1267vs 43 ls 498vs
9. 1613m 1596vs 1276vs 442vs 571m
615m,540m
260m,222s
10. 1615m 1596vs 1272s 421vs 560s 590s,530m
11. 1630m 1602vs 1250vs
12. 161 lm 1602vs 1262vs 444m 535s 268s,232m
13. 1607m 1601vs 1259s 43 lm 505s 605s,560vs
14. 1612m 1602vs 1271vs 440s 569vs 260m,228m
15. 1614m 1602vs 1273m 434s 565vs 595m,535m
*, SI.Nos. are those as indicated in Table I; m, medium; vs, very strong; s, strong.
tra of the complexes suggesting the deprotonation of the phenolic OH group on complex for-
mation. The appearance of azomethine vC--N vibrations in all the Schiff bases at lower fre-
quencies (1632 + 2 cm) in comparison with the normal position (1675 cm-) indicates the
involvement of the azomethine nitrogen atoms in hydrogen bonding (Fig. II). This band suf-
fers a negative shift (1615 + 8 cm-) on complex formation suggesting coordination of the
azomethine nitrogen to tin. Other characteristic IR bands observed in the spectra of the Schiff
bases at 1599 +/- 3, 1022 + 2 and 676 +/- 2 cm
-have been assigned (9) to the v >C--N--
N--C< (cyclic), vN--N and vC-S-C modes .of vibrations, respectively, of the thiadiazole
302
M. Nath andS. Goyal Metal-Based Drugs
ring. In the spectra of all the complexes these vibrations remain almost unchanged indicating,
thereby, non-involvement of the ring nitrogen and sulphur in coordination. The IR spectra
of the Schiff bases display a band at 1254 + 4 cm,characteristic of the phenolic vC--O
vibration undergoes a positive shift in the spectra of the complexes indicating coordination
Fig. II: Hydrogen bonded structure of (LH-1).
of the Schiff bases through the phenolic oxygen atom. The coordination through the oxygen and
nitrogen is further supported by the occurrence of new bands at 535 + 37 and 428 + 16 cm
-in
the spectra of the complexes which may be assigned (10) to vSn-O and vSn---N, respectively.
The far-IR spectra of Ph3SnL and Ph2SnL2
show bands at 265 4- 6 and 229 + 7 cm
-which
may be assigned (7,11) to the v,,Sn-C and v,Sn-C, respectively, whereas the corresponding
bands at 608 + 18 and 550 + 20 cm
-have also been assigned (10) in the spectra of Me3SnL
and BuzSnLz.
H NMR Spectra
The conclusion drawn from ]H NMR spectral studies lend further support to the mode of bond-
ing discussed above. The H chemical shifts 5 (in ppm) of the Schiff bases and their complexes
are listed in Table IV. A signal at 8.50 + 0.20 ppm due to the intramolecularly hydrogen bonded
phenolic proton (8,9) of the Schiff bases disappear in the H NMR spectra of the organotin com-
plexes indicating, thereby, the substitution of the phenolic proton by the organotin moiety. The
signals due to the azomethine (-C(H)--N-) and methyl (-C(CH3)--N--) protons in the Schiff
bases appear as a singlet at 7.05 and 1.60 ppm, respectively indicating that the azomethine ni-
trogen is hydrogen bonded (8,9). In the complexes, the signals shift downfield (2.10 ppm for the
methyl and 8.20-8.50 ppm for the methine protons) as compared to their positions in the free
Schiff bases owing to the coordination of the azomethine nitrogen to tin (8,9). The signals at
4.57 + 0.03 ppm in the Schiff bases has been assigned to the --OCHa protons which remains
unaltered on complexation and thus clearly indicates the non-involvement of this group in com-
plex formation. The complex pattern observed in the region 7.00-8.25 ppm in all the complexes
is due to overlapping resonances of the phenyl groups bonded to tin and of the aromatic ligand
protons. The butyl protons attached to the tin appear as a complex pattern (1.70 0.83 ppm)
(12,13). The number of protons of various groups calculated from the integration curves and those
calculated for the expected molecular formula agrees with each other.
gSn Missbauer Spectra and Structure Determination
Q.S. (Quadrupole Splitting) and I.S. (Isomeric Shift) values of the complexes are presented in
Table V. The complexes viz., RaSnL (R CH or C6H5) and 1L2SnL2 (R CH_ or C4H9) are
five- and six-coordinated, respectively, having monofimctional bidentate ligands with ON donor
sites as indicated from the IR data. Trigonal bipyramidal R3SnL (L bidentate ligand) complexes
have been reported (11,14) to have different Q.S. for the following three isomers (Q.S. 1.7-2.3
mm s-]
for a; 3.0-3.9 mm s-!
for b and 3.5-4.1 nun s-1
for c in Fig. III). The observed values cf
303
Vol. 2, No. 6, 1995 SpectralStudies & Bactericidal, Fungicidal,Insecticidal
&ParasitologicalActivities ofOrganotin(llO Complexes
0
304
M. Nath andS. Goyal Metal-BasedDrugs
Q.S. (Table V) of the complexes, viz., Ph3Sn(L-2), Ph3Sn(L-3), and Me3Sn(L-3) suggest the struc-
ture (a).
From the observed Q.S. (1.91-2.56 mm s) for the complexes, RzSnL2 [R C6H or con9
L anion of (HL-1) to (HL-3)], the existence of cs-RSn(IV) moieties in a distorted octahe-
(a) (b)
Fig. III" Possible isomers of trigonal bipyramidai RSnL (L bidentate iigand)
dral arrangement around tin atom (Fig. IV) has been suggested which is also consistent with the
IR data.
k
Fig IV Structure of ISnL2 [R C6Hs or C4H9; L anion of (HL-1) to (HL-3)].
Table V: The Sn M6ssbauer Spectroscopic Data of Orlanotin{IV} Complexes
Complex Q.S. (mm s"a) I.S. (mm s-l)
Ph2Sn(L-1)2 1.91 + 0.05 0.97 +/- 0.01
Ph3Sn(L-2) 2.44 + 0.06 1.10 +/- 0.01
Ph2Sn(L-2)2 2.15 + 0.07 1.12 +/- 0.27
Ph3Sn(L-3) 2.19 + 0.05 1.16 + 0.01
Me3Sn(L-3) 2.82 + 0.04 1.15 A 0.01
PhzSn(L-3)z 2.56 + 0.04 1.27 + 0.01
Bu2Sn(L-3)2
2.09 + 0.05 0.99 + 0.01
Antimicrobial Activity
The antimicrobial activity data, compiled in Table VI, show that all the Schiff bases are active
against fungal strains 6 to 10 only. Among all the Schiff bases, (HL-2) was found to be the
305
Vol. 2. No. 6. 1995 SpectralStudies &Bactericidal, Fungicidal,Insecticidal
& ParasitologicalActivities ofOrganotin(IlO Complexes
0
V V V
V V V V V V V V
306
M. Nath andS. Goyal Metal-BasedDrugs
most active. The Schiff bases (HL-I) and (HL-2) were found to be active against fungal strains
6, 7, 8 and 10, and (HL-3) was active against fungal strain 7 only.
As evident from Table VI, diphenyltin complexes are very active against all the bacteria
and fungi used and show lower MIC values in comparison to the Schiff bases. The other com-
plexes also show greater bactericidal and fungicidal activities as compared to their corresponding
Schiff bases and show lower MIC values than those of the Schiff bases. The order of the activ-
ity is as follows: Ph2SnL2>Me3SnL>Ph3SnL>Bu2SnL2
Thus the results clearly indicate that the organotin(IV) complexes possess moderate bacte-
ricidal and fungicidal activities. The results of herbicidal, insecticidal, fungicidal and parasitologi-
cal activities of (HL-1) and (HL-2), and the organotin complexes derived from them (see Table
VII) indicate that all the compounds tested except Ph3Sn(L-2) are completely inactive against the
various species mentioned.
Table VII: Results of Biological Activities of the Schiff Bases and their Com-
plexes
Schiff base/
complex
(HL-I)
PhSn(L- )
Bu2Sn(L-1)2
(HE-2)
PhSn(L-2)
MeSn(L-2)
Bu2Sn(L-2)2
Herbicide Insecticide
Mini High Primary
Fungicide Animal Health
Mini Primary Nematode
q), Compound inactive; 5, Compound has some activity;
Herbicidal Species-Abutilon theophrastt', Alopecurus myosuroides, Ambrosia artemisiifofia, Avena
fatua, Brassica kaber, Bromus tectorum, Cyperus rotundus, Echinochola crus-galli, Elytrigia
repens, Galum aparine, Glycine max, lpomoea spp., Lolium multiflorum, Sesbama exaltata,
Setaria viridis, Sorghum halepense, Triticum aestivum, Zea mays
Insecticidal Species- Spodoptera eridania, Tetranychus urticae, Diabrotica undecimpunctata
Fungicidal Species (Mini screen)- Botrytis cinerea, Sphaerotheca fuliginea, Pseudoperonospora
cubensis, Rhizoctonia solani
Fungicidal Species (Primary screen) In vivo- Venturia inaequalis, Plasmopara viticola,
Botrytis cineria, Pyricularia oryzae, Cercospora beticola, Phytophthora infestans, Puccima
recondita, Puccinia gramminis
In vitro- Fusarium oxysporum, Pythium ultimum, Rhizoctonia solan, Pseudocercosporella sp.
Thermal Studies
Thermal decomposition of two complexes, viz., Ph3Sn(L-I) and Ph2Sn(L-2)z has been studied us-
ing TG, DTG and DTA tectmiques. Both complexes decompose gradually with the formation of
SnO: as an end product.
307
Vol. 2, No. 6, 199.$ Spectral Studies & Bactericidal, Fungicidal,Insecticidal
& ParasitologicalActivities ofOrganotin(IlO Complexes
308
M. Nath andS. Goyal Metal-BasedDrugs
As evident from Table VIII no sharp and distinct plateau was observed for the loss of the
ligand or organic groups attached to tin in the TG curve of Ph3Sn(L-1) in the temperature range
80-796C whereas DTA and DTG show three peaks at 120 (endothermic), 566, 722C (both exo-
thermic) and 107, 226, 739C, respectively. The total observed weight loss in TG (78.00%) cor-
responds to the formation of SnO as end product of the decomposition of the complex. The ob-
served 'd' values in the residue were 3.36, 2.58, 1.77 and 2.30 and Obsd: Sn was 78.69% whereas
the reported 'd' values in SnOz are 3.35, 2.64, 1.77, 2.37 (15).
As evident from Table VIII the first step of the decomposition of the complex PhSn(L-2)
extends from 51 to 521C and the observed weight loss (39.00%) as against the calculated value
(38.49%) corresponds to the loss of the thiadiazole moiety of the ligand (2 mole) giving
C3aH:,N:OzSn as imermediate (I) which is supported by elemental analyses (Obsd: C, 66.72; H,
3.87; Sn, 19.36%; Calcd: C, 66.81; H, 3.96; Sn, 19.42% for C3HzNzOzSn). It is also confirmed
by the disappearance of the characteristic frequencies of the thiadiazole ring in the infrared spec-
trum of the residue obtained by isothermal heating of the complex at 525 + 4C. The second
step of the decomposition corresponds to the loss of the organic groups attached to Sn and of
the ligand molecules giving SnOz as end product, which was characterized by powder X-ray dif-
fraction analyses and tin determination ['d' values Obsd: 3.33, 2.63, 1.79 and 2.33; Obsd: Sn,
78.78%].
REFERENCES
1. D.R. Williams, Chem. Rev., 72, 203 (1972).
2. R.S. Srivastava, L. D. S. Yadav, R. K. Khare and A. K. Srivastava, Ind. J. Chem.,
20A, 516 (1981) (References therein).
3. V.H. Shah, H. H. Patel and A. R. Parikh, J. lnd. Chem. Soc., 59, 678 (1982).
4. M. Nath and S. Goyal, Main Group Met. Chem., 16, 167 (1993).
5. M. Nath and S. Goyal, Main Group Met. Chem., 18, 51 (1995).
6. V. Ranga Rao and V. R. Srinivasan, Ind. J. Chem., 8, 59 (1970).
7. M. Nath, N. Sharma and C. L. Sharma, Synth. React. lnorg. Met. -Org. Chem., 20, 623
(1990).
8. K.C. Satpathy, B. B. Jal and R. Mishra, lnd. J. Chem., 25A, 196 (1986).
9. K.C. Satpathy, B. B. Jal and R. Mishra, lnd. J. Chem., 24A, 147 (1985).
10. R.C. Poller, Logos Press, London, (1970) (References therein).
11. L.E. Khoo, J. P. Charland, E. J. Gabe and F. E. Smith, lnorg. Chim. Acta., 128, 139
(1987).
12. R. Tiwari, G. Srivastava, R. C. Mehrotra and A. J. Crowe, lnorg. Chim. Acta, 111,
167 (1986).
13. A. Saxena and J. P. Tandon, Polyhedron., 3, 681 (1984).
14. G.M. Bancrot, B. W. Davies, N. C. Payne and T. K. Sham, J. Chem. Soc. Dalton,
973 (1975).
15. "Power diffraction file sets 1-10, joint committe on powder diffraction standards", Phila-
delphia, PA, P. 21 and 1-984 (1987).
Received: September 13, 1995 Accepted: October 2, 1995
Received in revised camera-format" November 9, 1995
309

				

